# Optimizing Dynamic Antibiotic Treatment Strategies against Invasive Methicillin-Resistant *Staphylococcus Aureus* Infections using Causal Survival Forests and G-Formula on Statewide Electronic Health Record Data

**Published:** 2023-08

**Authors:** Inyoung Jun, Scott A. Cohen, Sarah E. Ser, Simone Marini, Robert J. Lucero, Jiang Bian, Mattia Prosperi

**Affiliations:** Department of Epidemiology, College of Public Health and Health Professions, University of Florida, Gainesville, FL 32610, USA; Department of Epidemiology, College of Public Health and Health Professions, University of Florida, Gainesville, FL 32610, USA; Department of Epidemiology, College of Public Health and Health Professions, University of Florida, Gainesville, FL 32610, USA; Department of Epidemiology, College of Public Health and Health Professions, University of Florida, Gainesville, FL 32610, USA; School of Nursing, University of California, Los Angeles, Los Angeles, CA 90095, USA; Department of Health Outcomes and Biomedical Informatics, College of Medicine, University of Florida, Gainesville, FL 32610, USA; Department of Epidemiology, College of Public Health and Health Professions, University of Florida, Gainesville, FL 32610, USA

**Keywords:** Individualized Treatment Effect, Causal Machine Learning, Causal Survival Forest, G-Formula, Dynamic Treatment Optimization, Antibiotic Resistance

## Abstract

Developing models for individualized, time-varying treatment optimization from observational data with large variable spaces, e.g., electronic health records (EHR), is problematic because of inherent, complex bias that can change over time. Traditional methods such as the g-formula are robust, but must identify critical subsets of variables due to combinatorial issues. Machine learning approaches such as causal survival forests have fewer constraints and can provide fine-tuned, individualized counterfactual predictions. In this study, we aimed to optimize time-varying antibiotic treatment –identifying treatment heterogeneity and conditional treatment effects– against invasive methicillin-resistant *Staphylococcus Aureus* (MRSA) infections, using statewide EHR data collected in Florida, USA. While many previous studies focused on measuring the effects of the first empiric treatment (i.e., usually vancomycin), our study focuses on dynamic sequential treatment changes, comparing possible vancomycin switches with other antibiotics at clinically relevant time points, e.g., after obtaining a bacterial culture and susceptibility testing. Our study population included adult individuals admitted to the hospital with invasive MRSA. We collected demographic, clinical, medication, and laboratory information from the EHR for these patients. Then, we followed three sequential antibiotic choices (i.e., their empiric treatment, subsequent directed treatment, and final sustaining treatment), evaluating 30-day mortality as the outcome. We applied both causal survival forests and g-formula using different clinical intervention policies. We found that switching from vancomycin to another antibiotic improved survival probability, yet there was a benefit from initiating vancomycin compared to not using it at any time point. These findings show consistency with the empiric choice of vancomycin before confirmation of MRSA and shed light on how to manage switches on course. In conclusion, this application of causal machine learning on EHR demonstrates utility in modeling dynamic, heterogeneous treatment effects that cannot be evaluated precisely using randomized clinical trials.

## 1 Introduction

Antimicrobial resistance (AMR) is a global public health threat. Every year, there are over 2.8 million antimicrobial-resistant infections in the United States, and at least 35,000 people die from these infections (Centers for Disease Control and Prevention, 2019). Identifying optimal antibiotic treatment regimens is key to increasing the chance of favorable clinical outcomes for individuals, and it can also have a role in preventing AMR at the population level. The World Health Organization (WHO) and the US Centers for Disease Control and Prevention (CDC) consider methicillin-resistant *Staphylococcus aureus* (MRSA) infection to be a bacterial disease of priority concern, because MRSA can affect multiple tissues and organs, and its treatment options are few, due to the fact that it is resistant to nearly all beta-lactam antibiotics (Centers for Disease Control and Prevention, 2019; World Health Organization, 2017; Lee et al., 2018; Turner et al., 2019; Rodvold and McConeghy, 2014; Alexander et al., 2023).

Invasive MRSA is a life-threatening complication in which the infection has spread inside the body, e.g., the bloodstream, deep-seated skin, or lungs (Lee et al., 2018). With invasive infections, patients often need emergent antibiotic treatment before the pathogen causing the infection is known. This initial treatment is called ‘empiric treatment’. Clinicians use symptoms and source of infection to select empiric treatment according to experience and international guidelines (VanEperen and Segreti, 2016). Once the pathogen is identified by test results, clinicians can prescribe the first directed treatment. Currently, a bacterial identification culture test takes 2 – 3 days. Because of this time delay from testing, in clinical practice, several sequential treatment scenarios are possible for each patient (Jeffres, 2017; Leekha et al., 2011).

Vancomycin is known as the first-line treatment for patients with a suspected MRSA infection (VanEperen and Segreti, 2016; Levine, 2006). Even though vancomycin has broad coverage, there can be multiple reasons to change vancomycin dose or switch to other antibiotics, including a lack of clinical improvement, updated information regarding antibiotic susceptibility, development of adverse effects, and even financial reasons (Jeffres, 2017). Nephrotoxicity is one of the main adverse events that patients experience while receiving vancomycin. When a patient experiences renal toxicity, doctors prescribe alternative antibiotics (Liu et al., 2020; Sawada et al., 2018; Filippone et al., 2017).

Although a randomized clinical trial (RCT) can be ideal approach for comparing first directed treatment for known drug susceptibility, this approach is cumbersome for dynamic, i.e., time-varying, antibiotic treatments especially in the context of delayed pathogen culture and antibiotic susceptibility testing. It is even more challenging to conduct a RCT with MRSA. MRSA must be treated immediately under multifaceted conditions. Estimating dynamic, heterogenous treatment effects among different patient groups is difficult to establish using a RCT, requiring a complex design and larger sample. However, observational data could be leveraged to calculate dynamic individualized treatment effects since numerous treatment scenarios are possible and observed in practice.

Previous studies of MRSA patients have compared vancomycin to other antibiotics, including daptomycin and linezolid (Schweizer et al., 2021; Yue et al., 2016; McCreary et al., 2020; Yeager et al., 2021; Paul et al., 2015; Liang et al., 2014). In a retrospective study of veterans with MRSA bloodstream infections, patients who were switched from vancomycin to daptomycin during the first three days after starting treatment had a lower risk of 30-day mortality than patients who did not switch from vancomycin (Schweizer et al., 2021). When compared to vancomycin, linezolid has shown promise for treating patients with skin and soft tissue infections (Yue et al., 2016) and ventilator-associated pneumonia (Peyrani and Ramirez, 2015).

Overall, there is a lack of studies that looked specifically at dynamic sequential treatment effects. To our knowledge, none applied methods for heterogeneous effect estimation. In this work, we analyzed longitudinal electronic health record (EHR) data from a large, statewide hospital settings, together with causal survival forest and g-formula, to estimate both average effects and heterogeneity of conditional effects for dynamic antibiotic therapy –vancomycin vs. others– on mortality in patients admitted with invasive MRSA infection.

## 2 Methods

First, we describe our ethical approvals, the data source, the study population, the longitudinal design, and the sequential treatment strategies, outcome, and covariates. Second, we give an overview of the causal survival forest and the g-formula methods, which we used to estimate dynamic treatment effects.

### 2.1 Ethics Statement, Data Source, and Derivation of Study Population

As authors, we abide to the ethical principles for medical research involving human subjects outlined by the World Medical Association in the Declaration of Helsinki. This study was reviewed and approved by the University of Florida’s (UF) institutional review board (IRB) (protocol number IRB 201900652). We used deidentified data from a large university hospital system in Florida, UF Health, that comprises two primary hospitals in Gainesville and Jacksonville, as well as forty-five outpatient clinics in the state. Since 2011, UF Health uses the Epic system (https://www.epic.com/), and the EHR data is warehoused in the Integrated Data Repository (IDR, https://idr.ufhealth.org/). The IDR includes patients’ demographics, clinical diagnoses, procedures, laboratory tests, and medications. Clinical diagnoses and procedures are encoded using the International Classification of Disease (ICD, https://www.who.int/standards/classifications/classification-of-diseases) ontology, 9th and 10the revision, while laboratory tests and medications are encoded via the Logical Observation Identifiers Names and Codes (LOINC, https://loinc.org/) and the RxNorm (https://www.nlm.nih.gov/research/umls/rxnorm/index.html) terminology, respectively. Data requests made to the IDR staff (https://idr.ufhealth.org/research-services/) should be in compliance with institutional, state and Federal regulations. The authors of this work are willing to share the study protocol and data analysis code.

**Figure 1:**
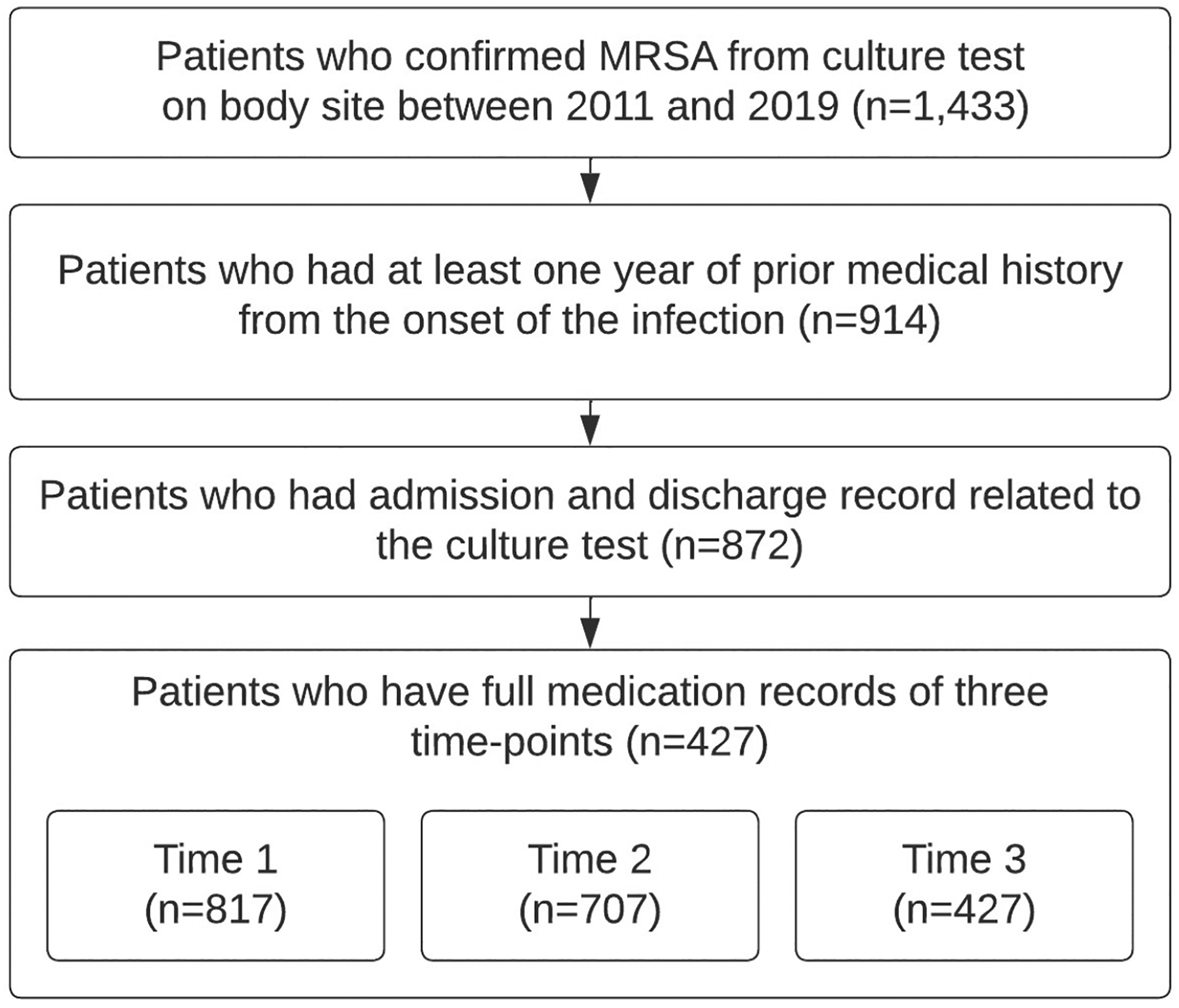
Flowchart of inclusion criteria to derive the study population

Our study population includes adults (18 years and older) admitted to the hospital and diagnosed with invasive MRSA (the first one recorded). An MRSA diagnosis was confirmed with a culture test based on a biological sample, including blood, fluid, bone, kidney, liver, heart, lung, pancreas, etc. Individuals who had at least one-year of medical records before the identification of MRSA were included in this study to account for relevant medical history. Patients were followed during three sequential time points: (1) empiric treatment, (2) possible switch to the first directed treatment, and (3) the sustaining therapy with other switching options. The flowchart of the inclusion criteria for the study population is given in Figure 1.

### 2.2 Three Timepoints for Constructing Sequential Strategies

**Figure 2:**
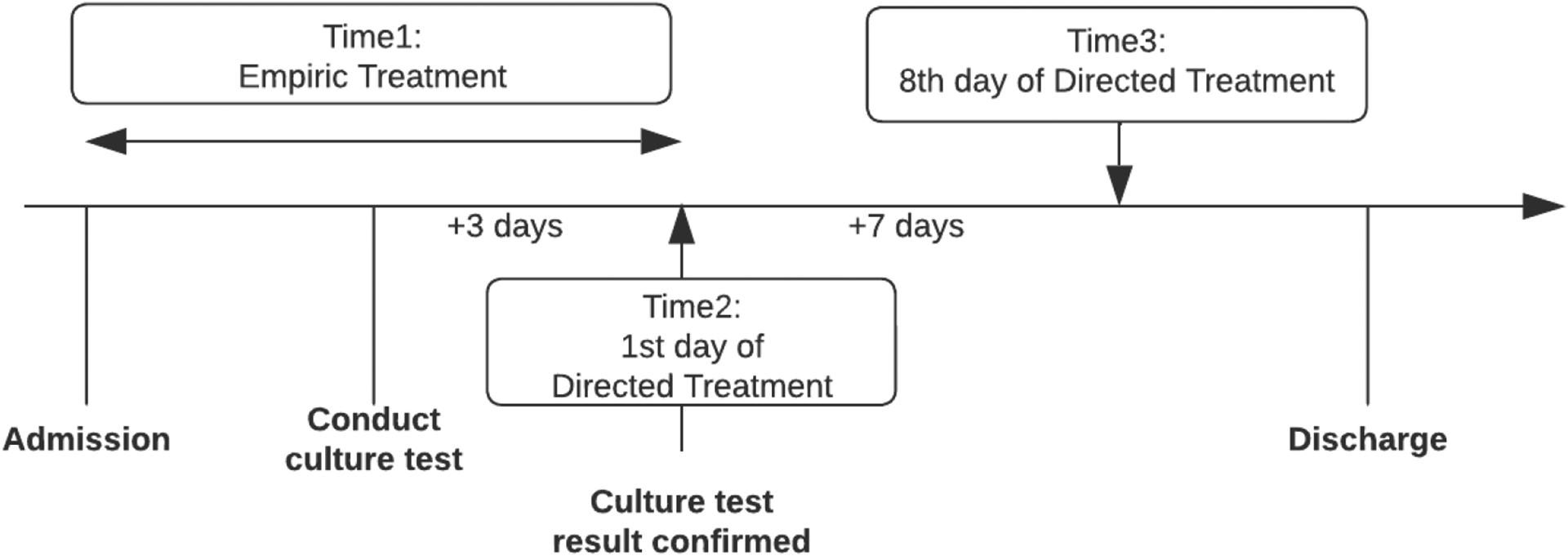
Description of treatment timeline and three timepoints Note: T1 is an interval period from the start of admission to the culture test result confirmed, T2 is the 1st day of the directed treatment, and T3 is the 8th day of directed treatment

Selecting appropriate timepoints is crucial not only for assessing the sequential treatment effects, but also for identifying when treatment changes can be acted upon. We focused on the sequence of (1) empiric, (2) empiric to directed, and (3) sustaining treatment assignments as illustrated in Figure 2. “Time 1” refers to the interval period from admission to the receipt of culture test results. For example, if a patient received vancomycin during this interval, we labeled the patients into vancomycin group at Time 1. During this time, the definitive organism and antibiotic susceptibility test results are not known. We collected the relevant measurement proxies from the EHR to ascertain the treatment propensity, as well as potential causes of early/late adverse reactions that can entail contraindications. “Time 2” is a measure of the preliminary response to the empiric antibiotic therapy, also known as early response assessment. Since the initial response period is typically assessed within 3 – 7 days, we fixed it at 3 days after the culture test. With these results, providers may continue with their empiric treatment prescription (perhaps with dose adjustment) or switch to the directed treatment. Various clinical factors, such as nephrotoxicity, may affect this transition. “Time 3” involves monitoring the antibiotic treatment and sustaining therapy for the recommended time. For MRSA, this is typically between 7 and 14 days after the initiation of therapy (or even longer) depending on the severity and location of the infection. During this time period, the overall effectiveness of the therapy, any remaining signs of infection, and the potential for recurrence or complications is assessed. In our study, we define this third time point as the 7 days from the first directed treatment (i.e., “Time 2” + 7 days, or “Time 1” + 10 days).

At each of the three timepoints, we assessed if the patient was taking vancomycin or they were prescribed another. In total, 8 different sequential treatment strategies were considered. For example, if a patient started with vancomycin as empiric treatment and maintained the same treatment at timepoint 2, but then changed to another antibiotic at timepoint 3, the patient would have ‘1-1-0’ as the value for the sequential treatment strategy variable.

### 2.3 Study Intervention, Outcome, and Covariates

**Figure 3:**
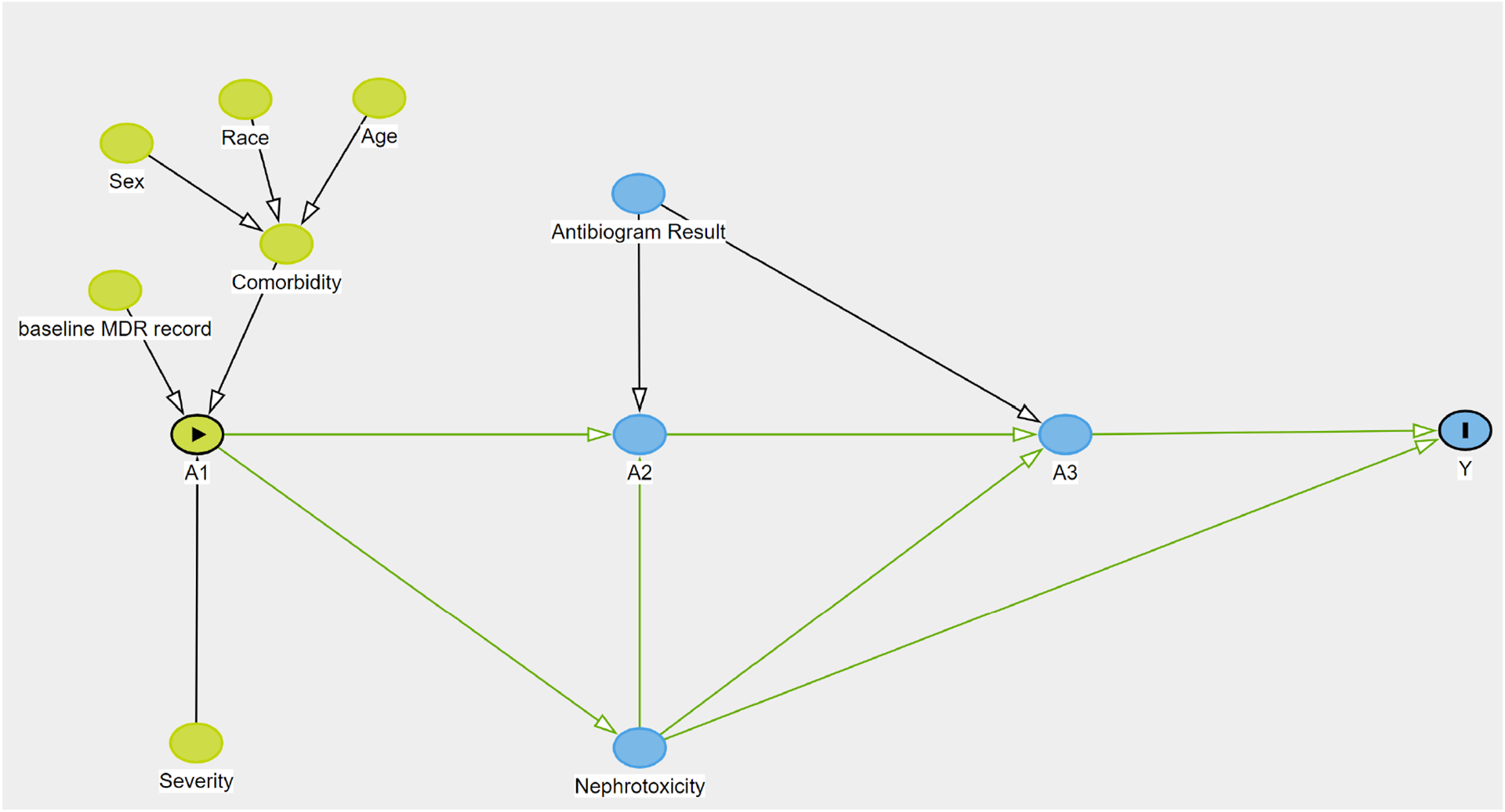
The directed acyclic graph representing causal relationships among sequential antibiotic treatment (A1 at Time 1, A2 at Time 2, and A3 at Time 3), influencers and confounding factors, and 30-day mortality (Y) Note: A1, A2, A3 are treatment variables at each time point (either vancomycin or others). Y is a 30-day mortality. Demographics, comorbidity, severity, and past multi-drug resistance from previous EHR records are measured at baseline. Nephrotoxicity is a time-varying confounder that affects the following antibiotic prescriptions (A2, A3). In this figure, we included known risk factors between antibiotic treatment and mortality.

We defined two different interventions that could be applied in clinical practice: one was a three-point treatment sequence (modelled using a causal survival forest) and the other was a treatment update at a given time point (modelled through g-formula). For the first intervention, we encoded a binary treatment variable to indicate whether there was an antibiotic change between the previous time point to next time point (i.e., treatment change from Time 1 to Time 2 or treatment change from Time 2 to Time 3, and any change between the first and another time point, i.e., from Time 1 to Time 2/Time 3). For the second intervention, vancomycin was the target treatment, and any other antibiotic was pooled into the control group, corresponding to the 8 sequential treatments. The study outcome was the time from the onset of bacterial infection (i.e., culture collection date set as the index date) to death or discharge within a 30-day horizon (i.e., 30-day mortality). Study covariates were both time-fixed for the first intervention and time-varying for the second intervention. Time-fixed covariates measured before MRSA onset or at index date included patient’s demographics (age, sex, race), Charlson’s comorbidity index, admission type, intensive care unit (ICU) stay, healthcare acquired infection, and previous antimicrobial resistance testing. We also collated all prior clinical diagnoses present with at least 10% frequency in the study population (mapping all ICD-10 codes into ICD-9), to investigate additional potential drivers of the outcome, as done in another study (Jun et al., 2022). The setup for time-varying covariates collected after the index date (time-fixed) is illustrated in Figure 3 using a causal directed acyclic graph (DAG). For example, one time-varying confounder is the nephrotoxicity variable, defined as a 50% decrease in creatinine clearance (CrCl) from a baseline value (Wong-Beringer et al., 2011). If a patient was missing the creatinine clearance value at a given time point, the previous creatinine value closest to the time point was used.

### 2.4 Causal Survival Forests and G-formula

Causal survival forests (CSF) are an adaptation of the causal forest algorithm, a nonparametric method for estimating heterogeneous treatment effects in survival settings with right-censored data (Cui et al., 2023). The causal effect of the antibiotic sequential strategy is estimated under the Neyman-Rubin’s potential outcome model framework (Imbens and Rubin, 2015). This estimation operates within a statistical setting where we have n independent and identically distributed subjects (*i* = 1*, … , n*), and we observe each subject’s tuple (*X*_*i*_*, Y*_*i*_*, W*_*i*_*, D*_*i*_).

In this context, *X*_*i*_ denotes a vector of covariates, *Y*_*i*_ represents the observed response variable (i.e., days to death), *W*_*i*_ *∈ {*0, 1*}* signifies the binary treatment assignment (specifically, whether or not a change in the antibiotic was made), and *D*_*i*_ acts as an event indicator, signifying whether the event (i.e., death) took place. Given this configuration, we can identify (1) if there are heterogeneous effects among patients and (2) which specific population (i.e., which combination of covariates) shows high heterogeneity. We have employed the concepts of the Conditional Average Treatment Effect (CATE) and the Rank-Weighted Average Treatment Effect (RATE) to quantify these heterogeneous effects.

The CATE is defined by the equation *CATE* = *E*[*Y*_*i*_(1) − *Y*_*i*_(0)*| X*_*i*_ = *x*] and is the expected mean of difference between potential outcomes *Y*_*i*_(0), *Y*_*i*_(1) given auxiliary covariates *X*_*i*_. CATE can be used to derive treatment prioritization rules, and the RATE serves the purpose of evaluating how good treatment prioritization rules are at distinguishing sub-populations with different treatment effects, or whether there exists notable heterogeneity. RATE only considers ranking of each patient’s rather than considering numeric size of the score. For quantifying the treatment benefit, the Targeting Operator Characteristics (TOC) is further calculated.

$$TOC(q)=E[Y_{i}(1)-Y_{i}(0)|S(X_{i})\geq F_{S(X_{i})}^{-1}(1-q)]-E[Y_{i}(1)-Y_{i}(0)]$$

In the TOC equation, *TOC*(*q*) implies the top q-th fraction of individuals with the largest prioritzation score *S*(*X*_*i*_). *F*_*S*(*Xi*)_ is the distribution function of *S*(*X*_*i*_) for comparing the ATE in the top q-th fraction of individuals with the largest prioritization score *S*(*X*_*i*_) with the overall ATE from treating everyone. If the TOC is equal to 0, it means that there is no benefit in stratifying the treatments using given prioritization rules. The parametric g-formula is an extended version of standardization by Robins for time-varying treatments and confounders. It uses the identification assumptions of inverse probability weighting, but it models the outcome means instead of the treatment equation (Ezzati et al., 2004; Westreich et al., 2012; McGrath et al., 2020). All analyses were conducted using the R software (https://www.r-project.org/), including the ‘grf’ (Tibshirani et al., 2023) and ‘gfoRmula’ (McGrath et al., 2020) packages.

## 3 Results

### 3.1 Population Characteristics, Outcomes, and Dynamic Treatment Assignments

Among 1,433 patients admitted between 2011 and 2019, with a confirmed MRSA diagnosis, 914 had at least one year of prior medical history from the onset of the infection and 872 patients had complete record data from admission to discharge. Of these, Time 1 was observed in 817 patients, Time 2 in 707 patients, and Time 3 in 427 patients. As the objective was to reach the sustained treatment time point, the final study population comprises the last subset of 427 patients.

The mean age of the study population was 55 years, 48.9% were male, 60.4% white, and 34.4% had multi-drug resistance (more than three antibiotic classes). The proportion of patients who stayed in the ICU was 52.2% and 24.1% were assumed to have an healthcare-acquired infection. The overall length of admission was median 19 days, from a minimum of 10 days to a maximum of 1,050 days. Out of the 427 patients, 33 patients (7.7%) died within 30 days from the MRSA onset. The summary statistics on the study population are given in Table 1.

Among the 427 patients, 96.9% started with vancomycin, 56.2% used vancomycin treatment throughout all three timepoints, while 43.8% of them changed from vancomycin to another antibiotic at least once; 21.3% changed their treatment from Time 1 to Time 2 and 32.6% from Time2 to Time 3. In Table 2 we summarized the number of subjects within each possible scenarios of antibiotic treatment at the three sequential timepoints corresponding to the empirical, directed, and sustaining treatment periods, along with the proportions of suspected nephrotoxicity, which is one of the main reasons for antibiotic change.

### 3.2 Estimation of Treatment Effects

For the first intervention, we generated two estimates of the conditional average treatment effect using the CSF. Model 1 included the expert-selected variables of the DAG (sex, race, age, Charlson’s comorbidity score, ICU stay, and multidrug resistance). The average treatment effect for changing antibiotics during any timepoint showed a reduction in the probability of death (Mean = −0.07619, SE=0.02953). All covariates had no effect on mortality (i.e., p-value below 0.05). Model 2 also included the DAG variables, but expanded all individual comorbidities. The average treatment effect of this model showed a negative effect (Mean = −0.0810, SE=0.02953) similar to Model 1. Table 3 details CATE results using the best linear predictor for both Model 1 and Model 2.

**Figure 4:**
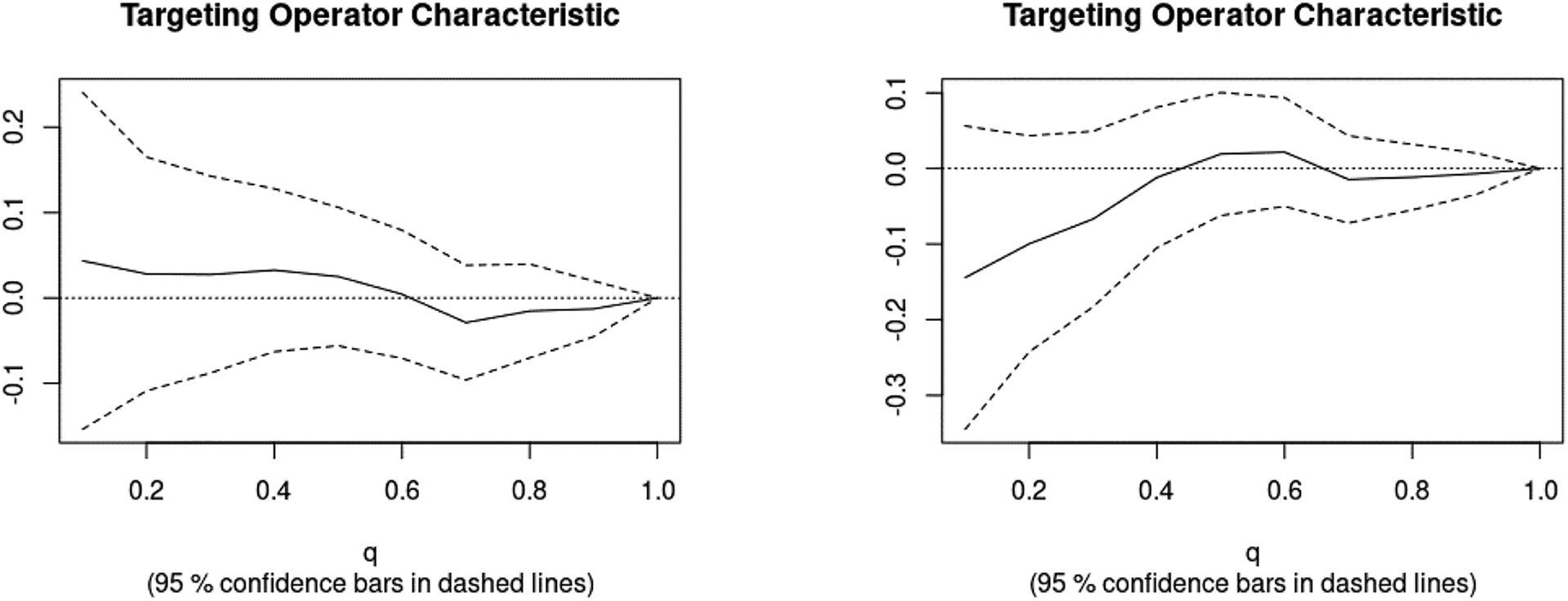
Targeting Operator Characteristics Curves for CSF-based CATE models: Model1 depicted on the left side and Model2 depicted on the right side graph

We then assessed heterogeneity for the CSF models using the area under the TOC curve (AUTOC) from the RATE function (Figure 4). In Model 1, there was minimal heterogeneity of treatment effect (AUTOC = 0.01, S.E = 0.06). In Model 2, the group with quantile above 15 would benefit from the treatment change (AUTOC = −0.04, S.E = 0.06).

In the analysis of the second intervention, we used the g-formula to estimate the effect of vancomycin vs. other treatment updated at each time point on 30-days mortality, in the presence of time-varying and time-fixed confounding. Table 4 shows the g-formula mean, ratio, and difference for the reference Intervention 0, which was the observed treatment (natural course), compared to the Intervention 1 scenario of “Never treat with vancomycin or switch immediately after empiric treatment”, and Intervention 2 scenario of “Always treat with vancomycin and do not switch to another antibiotic”. The mean ratio and mean difference highlight the difference between intervention scenarios using observed scenario as a reference group. For the whole study population, under the reference scenario, the empirical risk of the outcome was 7.7%, corresponding to the event proportion (30-day mortality) in our study population. If all patients were assigned to Intervention 1, the risk increased approximately to 14.6%. If all patients were assigned to Intervention 2, the risk was lower than the observed treatment scenario which was about 5.0%.

In the subset of patients who started their empiric treatment with vancomycin, under Intervention 1, the risk increased to 16.7% from the reference scenario. Under Intervention 2, the risk was 4.97%, lower than the observed treatment scenario. Overall, results in the whole and vancomycin-empiric population were similar, and the current sequential vancomycin treatment was better than not giving anyone vancomycin, but worse than giving everyone vancomycin.

## 4 Discussion

We found that switching from vancomycin to another antibiotic improved survival probability. Additionally, there was benefit from initiating vancomycin compared to not using it at any time point. Our findings are consistent with the general knowledge of clinical efficacy of vancomycin in the treatment of invasive MRSA infections obtained through RCTs. In our population, over 95% of patients were prescribed empiric vancomycin treatment, reflecting the common preference for this antibiotic in managing potential infections that have not yet been confirmed (VanEperen and Segreti, 2016). However, despite being the first-line choice of treating invasive MRSA infections, vancomycin was consistently used during all three timepoints in only half (56%) of the of the population and many patients who initially treated with vancomycin switched to another antibiotic at least once. This change might suggest concerns over vancomycin’s side effects, particularly nephrotoxicity, from the provider (Jeffres, 2017), reflecting the importance of considering these time-varying components in our models. This is also supported by the increased prevalence of nephrotoxicity in those who received consistent vancomycin treatment. Despite this, we found a decreased mortality probability when patients stayed on vancomycin, emphasizing its role despite potential complications.

In addition to time-varying treatment effects, we investigated putative treatment heterogeneity, which is key to the development of personalized treatments that cater to individual patient characteristics (Varadhan et al., 2013). We found low evidence of heterogeneity, although it could have been due to lack of power. Lack of power is even more problematic with RCTs, that can become cumbersome and resource-heavy to include sufficiently diverse populations. Furthermore, RCTs can include inherent selection bias because of strict inclusion criteria. For example, those with establish renal failure are typically excluded from vancomycin trials (Paul et al., 2015), however these individuals remain a priority group in assessing effects of vancomycin due to known side effects of the antibiotic.

Our study has a number of limitations. Firstly, our study design and data analysis make simplifications (albeit clinically reasonable) in the treatment staging and decision-making process that are a conceptual abstraction. The timepoints utilized in this study do not exactly align with real clinical settings and patient populations. While culturing methods remain the preferred method for confirming MRSA infections, different institutions may implement other approaches that would deviate from the timeline we defined. It is important to note here that, even if the measurement time points are the same across all patients, there can be also chance of including immortal time bias which differs among treatment paths. Secondly, our analysis does not consider the dosage of vancomycin therapy. This is particularly important to take into account in future analyses, due to its relationship with nephrotoxicity that influence on antibiotic selection. Thirdly, we considered all MRSA infections in this analysis and did not differentiate between specific types of infections (e.g., bloodstream, lung, skin and soft tissue). It is possible that the relationship between sequential vancomycin therapy and outcomes may vary between anatomic sources of infections due to the inherent pharmacodynamics of vancomycin.

As statistical methodologies continue to advance, promising opportunities arise for future research in sequential treatment optimization, particularly through the incorporation of algorithms from causal inference and machine learning. Examples include the flexible Bayesian Additive Regression Trees (BART) and Counterfactual Regression (CFR), which adaptly handle high-dimensional environments and intricate non-linear relationships, respectively (Hill, 2011; Shalit et al., 2017). Despite not being originally designed for time-varying treatments or confounders, innovative variations of these models have been developed (Linero and Zhang, 2022). In our study, we focused on sequential time-varying treatment options and confounders, utilizing the G-formula due to its ability to manage complex, dynamic scenarios. However, evaluating a broader array of models could further substantiate our research findings.

## 5 Conclusion

In this study we operationalized sequential treatment strategies aimed at identifying relevant heterogeneity and optimizing risk based on individual patients’ characteristics. We demonstrated the utility of applying causal machine learning to real-world data within a framework that can be used to screen multiple intervention hypotheses, especially for life-threatening conditions, and select the most promising to be tested with conventional RCTs, possibly saving resources and lives.

## Figures and Tables

**Table 1:** Baseline characteristics of the vancomycin started groups and overall population

Variables*	Group				
	*V*_111_	*V*_110_	*V*_101_	*V*_100_	Overall
	(N=240)	(N=88)	(N=38)	(N=48)	(N=427)
*Patient’s demographics*					
Age	54.0(16.2)	57.1(18.6)	58.3(13.6)	57.1(12.5)	55.0(16.3)
Sex - Male	117(48.8%)	48(54.5%)	18(47.4%)	18(37.5%)	209(48.9%)
Race - White	160(66.7%)	49(55.7%)	21(55.3%)	19(39.6%)	258(60.4%)
*Medical conditions at or before index date*					
Diabetes	58(24.2%)	23(26.1%)	4(10.5%)	9(18.8%)	96(22.5%)
Renal Disease	95(39.6%)	45(51.1%)	26(68.4%)	39(81.3%)	211(49.4%)
Chronic pulmonary disease	128(53.3%)	47(53.4%)	24(63.2%)	29(60.4%)	237(55.5%)
Mild liver disease	71(29.6%)	23(26.1%)	9(23.7%)	10(20.8%)	117(27.4%)
Moderate/Severe liver disease	20(8.3%)	8(9.1%)	8(21.1%)	4(8.3%)	40(9.4%)
Cancer (any malignancy)	31(12.9%)	15(17.0%)	7(18.4%)	8(16.7%)	62(14.5%)
Peripheral vascular disease	100(41.7%)	33(37.5%)	24(63.2%)	25(52.1%)	183(42.9%)
Charlson’s Comorbidity Index	5.80(3.65)	6.52(4.48)	8.50(3.75)	7.42(3.30)	6.35(3.88)
*Evidence of antimicrobial resistance (infections prior to MRSA)*					
Aminoglycosides	33(13.8%)	19(21.6%)	13(34.2%)	12(25.0%)	79(18.5%)
Beta-lactams	114(47.5%)	42(47.7%)	23(60.5%)	28(58.3%)	214(50.1%)
Carbapenems	14(5.8%)	7(8%)	6(15.8%)	6(12.5%)	34(8%)
Fluoroquinolones	55(22.9%)	29(33.0%)	19(50.0%)	23(47.9%)	130(30.4%)
Glycopeptides	16(6.7%)	7(8%)	5(13.2%)	7(14.6%)	37(8.7%)
Polypeptides	3(1.3%)	1(1.1%)	0(0%)	1(2.1%)	5(1.2%)
Sulfonamides	54(22.5%)	25(28.4%)	14(36.8%)	14(29.2%)	111(26.0%)
Tetracyclines	24(10.0%)	13(14.8%)	9(23.7%)	8(16.7%)	55(12.9%)
Multi-drug resistance (3+)	66(27.5%)	33(37.5%)	21(55.3%)	21(43.8%)	147(34.4%)
*Admission information*					
ICU stay	133(55.4%)	37(42.0%)	26(68.4%)	25(52.1%)	223(52.2%)
Healthcare acquired	60(25.0%)	13(14.8%)	14(36.8%)	15(31.3%)	103(24.1%)
Days of admission	20[10,392]	19[10,115]	19[10,1050]	19[10,65]	19[10,1050]
*Outcome*					
Death within 30 days	11(4.6%)	11(12.5%)	5(13.2%)	5(10.4%)	33(7.7%)

* Mean(SD) or Median[Min,Max] or Count(%)

**Table 2:** Number of study subjects within each possible scenarios of antibiotic treatment (*A*_1_ *A*_2_ *A*_3_) at the three sequential timepoints corresponding to the empirical, directed, and sustaining treatment periods (*T*_1_ *T*_2_ *T*_3_), together with the proportion of suspected nephrotoxicity that can trigger antibiotic change.

#	*A*_1_	*A*_2_	*A*_3_	Overall	Suspected Nephrotoxicity	
					Yes(N=28)	No(N=39)
Time	*T*_1_	*T*_2_	*T*_3_	*T*_1_&*T*_2_&*T*_3_	*T*_1_ − *T*_2_	*T*_1_ − *T*_3_
1	Vancomycin	Vancomycin	Vancomycin	240(56.2%)	15(53.6%)	18(46.2%)
2	Vancomycin	Vancomycin	Others	88(20.6%)	7(25.0%)	8(20.5%)
3	Vancomycin	Others	Vancomycin	38(8.9%)	1(3.6%)	6(15.4%)
4	Vancomycin	Others	Others	48(11.2%)	4(14.3%)	5(12.8%)
5	Others	Vancomycin	Vancomycin	3(0.7%)	0(0%)	0(0%)
6	Others	Vancomycin	Others	2(0.5%)	0(0%)	0(0%)
7	Others	Others	Vancomycin	2(0.5%)	0(0%)	0(0%)
8	Others	Others	Others	6(1.4%)	1(3.6%)	2(5.1%)

**Table 3:** Estimate of the conditional treatment effect of changing antibiotics during any timepoint on to mortality, using the causal survival forest method.

CATE Model (1) - Using only DAG variables			
Average Treatment Effect: −0.07619 (SE = 0.02953)			
Individual Treatment Effect: Min: −0.1584, Mean: −0.0755, Median: −0.0739, Max: −0.0217			
Variables	Estimate	Std. Error	t value *Pr*(> |*t*|)
Sex	0.0407	0.0604	0.5004
Race	−0.0565	0.0580	0.3305
Age	−0.0008	0.0018	0.6429
Charlson’s Comorbidity Index	−0.0031	0.0073	0.6725
ICU stay	−0.0656	0.0569	0.2492
Multi-drug resistance	0.0627	0.0575	0.2760
CATE Model (2) - Using DAG + individual comorbidities			
Average Treatment Effect: −0.0810 (SE = 0.0281)			
Individual Treatment Effect: Min: −0.1083, Mean: −0.0782, Median: −0.0783, Max: −0.0462			
Variables	Estimate	Std. Error	t value *Pr*(> |*t*|)
Sex	0.0404	0.2723	0.8824
Race	−0.1513	0.3784	0.6903
Age	−0.0117	0.0110	0.2928
Myocardial infarction	−0.1721	0.4967	0.7299
Congestive heart failure	0.2024	0.3747	0.5906
Cerebrovascular disease	0.2644	0.4104	0.5212
Dementia	0.4225	0.6213	0.4985
Chronic pulmonary disease	0.3245	0.3663	0.3783
Rheumatoid disease	−0.2275	0.6859	0.7410
Peptic ulcer disease	0.372	0.4869	0.4471
Mild liver disease	−0.1906	0.2974	0.5234
Diabetes without complications	0.2187	0.6174	0.7241
Diabetes with complications	0.8421	0.7525	0.2664
Hemiplegia or paraplegia	0.1629	0.4264	0.7034
Renal disease	−0.0713	0.3952	0.8572
Cancer (any malignancy)	0.2896	0.4059	0.4776
Moderate or severe liver disease	−0.2539	0.6188	0.6827
Metastatic solid tumor	0.2895	0.5006	0.5647

**Table 4:** G-formula estimation the effect of vancomycin vs. other treatment updated at each time point on 30-days mortality, in the presence of time-varying and time-fixed confounding, stratified by empiric treatment (Time 1).

Population	Intervention	G-formula mean	Mean ratio	Mean difference
All patients (N=427)	0 – Observed Treatment	0.07645	1.00 (ref)	0.0000
	1 – Never treat with vancomycin or switch immediately after empiric treatment	0.14615	1.91	0.0696
	2 – Always Treat with vancomycin and do not switch to another antibiotict	0.05005	0.65	−0.0264
Patients received vancomycin at T1 (N=414)	0 – Observed Treatment	0.07533	1.00 (ref)	0.0000
	1 – Never treat with vancomycin or switch immediately after empiric treatment	0.16744	2.22	0.0921
	2 – Always Treat with vancomycin and do not switch to another antibiotict	0.04977	0.66	−0.0255
